# Wearable measured physiological stress and activity patterns changes after 2025 Los Angeles wildfire among older adults: findings from a pilot prospective cohort study

**DOI:** 10.1088/2752-5309/ae7faf

**Published:** 2026-07-10

**Authors:** Jiawen Liao, Ruoxue Chen, Rui Tian, Chenyu Qiu, Wu Chen, Zhenchun Yang, Jiayuan Hao, Yihui Ge, Yan Lin, Jessilyn Dunn, Michael Bergin, Frank D Gilliland, Marilyn Black, David Kalafut, Mark Wilson, Junfeng (Jim) Zhang, Zhanghua Chen

**Affiliations:** 1 Department of Population and Public Health Sciences, Keck School of Medicine of the University of Southern California, Los Angeles, CA, United States of America; 2 Department of Quantitative Health Sciences, Cleveland Clinic, Cleveland, OH, United States of America; 3 Nicholas School of the Environment, Duke University, Durham, NC, United States of America; 4 Department of Civil and Environmental Engineering, Duke University, Durham, NC, United States of America; 5 Biomedical Engineering Department, Duke University, Durham, NC, United States of America; 6 Biostatistics and Bioinformatics Department, Duke University, Durham, NC, United States of America; 7 Chemical Insights Research Institute (CIRI), Underwriters Laboratories Inc, Marietta, Georgia, United States of America

**Keywords:** wildfires, public health, wearable sensors, natural disaster

## Abstract

The wildland-urban interface (WUI) fires have adverse effects on both physical and mental health. However, early biosignals changes related to fires are not well understood. Digital wearables can capture real-time changes in physiological stress and activity patterns, providing insight into the mechanisms of health effects of WUI fires and identifying vulnerable populations during and after WUI fires. This study aimed to assess the immediate and sustained changes in physical activity patterns and physiological stress markers during and after the 2025 Los Angeles wildfires. We conducted a longitudinal study of 15 older adults (mean age 73.2 years) during the 2025 Eaton Fire in Los Angeles, using the digital Oura Ring (Gen 3) continuously. Data were collected at baseline (Dec 9th, 2024 to Jan 6th, 2025), during the fire (Jan 7th, 2025 to Jan 12th, 2025), and after the fire (Jan 13th, 2025 to Jan 27th, 2025). Daily activity patterns (physical activity and sleep duration) and physiological stress (sleep heart rate, heart rate variability, sleep fragmentation, and breath rate) were collected, and linear mixed-effects models were used to investigate how these biosignals changed and how evacuation alerts impacted these changes. During the fire, six out of fifteen participants received evacuation alerts. We observed a 41-minute increase in sedentary time (*P* = .007), a 34 min reduction in sleep duration (*P* = .002), and a 0.4 BPM increase in sleep breath rate (*P* = .009). Although activity patterns returned to baseline post-fire, markers of physiological stress, including sleep heart rate and breath rate, remained elevated. Among participants who received evacuation alerts, the immediate and prolonged impacts are larger. The changes in activity patterns and increases in physiological stress during and after the 2025 Los Angeles wildfire in this cohort can indicate potential health effects. Digital bio-signals may serve as early indicators of adverse health outcomes following a wildfire.

## 1. Introduction

Wildfires, particularly at the wildland-urban interface (WUI), pose significant risks to physical and mental health. The 2025 Eaton Fire in Los Angeles started on January 7th, 2025, prompted widespread evacuations [1], drastically deteriorate air quality [2–4], and raised concerns about immediate and prolonged health impacts among vulnerable populations, such as older adults. Previous studies have linked wildfire disasters and smoke exposure to adverse physical and mental health outcomes [5–11], and that elderly populations are particularly vulnerable [8, 12]. Similar results on adverse physical and mental health effects of wildfire have been found for the 2025 Los Angeles wildfires (Eaton and Palisades fires). One study also found increased outpatient and virtual cardiovascular and respiratory visits [13]; another study found higher odds of depression and PTSD among population who received evacuation alerts [14].

However, real-time impacts on physiological stress (i.e. sleep fragmentation, heart rate, heart rate variability (HRV), and breath rate) and activity patterns (i.e. physical activity and sleep duration) are unknown. The changes of these biosignals have been shown as early indicators of chronic diseases and mental health outcomes [15, 16] and may explain the mechanisms of WUI fires on human health. Several pathways linking WUI fires on human health, such as reduce physical activity and disturbe sleep, forced evacuations and increases of mental and physiological stress [9], can also be informed by capturing real-time data of these biosignals. We hypothesized that wildfire events would lead to immediate alterations in activity patterns and sustained increases in physiological stress. Furthermore, we hypothesized that due to the increased perceptions of stress associated with evacuation, the disturbance of daily activity and heightened physiological stress effects are greater among individuals receiving evacuation alerts.

## 2. Methods

### 2.1. Study design

The study participants were a subset of the original study subjects enrolled in an air purifier study (Trial Registration: NCT05867381) with a target sample size 112, only a small subset of target population was enrolled in this study due to the stepwise recruitment of the parent trial. This air purifier intervention study is a crossover trial, in which participants will receive air purifiers for 18 months, consisting of 9 months of true air filters and 9 months of sham air filters. The enrollment criteria included ages between 65 and 84 years, with a weight of no less than 50 kg (110 lb), living in Los Angeles County, and having a previous medical history of ischemic heart disease. All enrolled subjects are then invited to participate in this wildfire sub-study in 2024, which aims to assess the effectiveness of the air purifier during wildfire events. After enrolling in this study, participants will wear the Oura Ring (Gen 3, Oura Inc.), a commercially available wearable device, continuously for at least 6 months or until the main trial ends.

### 2.2. Data collection

During the follow-up of the wildfire sub-study, participants’ digital health markers were continuously collected by Oura Ring, which participants were asked to wear throughout the day and during sleep. The Oura Ring connects to the Oura App via Bluetooth on participants’ phones and synchronizes data remotely. The study team monitored the data quality daily and notified participants if data was missing every three days.

The Oura Ring equips an accelerometer to detect movement, activity intensity, and sleep-wake states, as well as a photoplethysmography (PPG) sensor to monitor heart and breath rate. For physical activity, the Oura Ring measures and calculates daily steps, daily activity minutes, and sedentary minutes. Sleep duration and sleep-wake patterns were estimated for each sleep period, including long sleep (main sleep), nap, and short sleep. The Oura Ring assessed heart rate and HRV by extracting features from PPG sensors, which were sampled at 250 Hz. The HRV was calculated using time-domain parameters based on the root mean square of the successive differences in heartbeat intervals at 5 min resolution during the main sleep period. Previous studies have validated the Oura Ring’s measures of steps and resting time [17], sleep and sleep-wake transitions [18], heart rate, HRV, and breath rate [19]. In this study, we utilized daily-level physical activity data, including daily low-intensity activity time, sedentary time, total sleep time, and step count, as well as daily physiological stress markers, such as sleep-wake transition times, sleep heart rate, breath rate, and HRV. We did not include moderate-intensity and high-intensity activities since all of our participants are from an older population with existing ischemic heart disease, leading to very limited time for moderate- and high-intensity activities. Data collected 30 d before 2025 Los Angeles wildfire started between Dec 9th, 2024 and Jan 6th, 2025 were categorized as before fire baseline; data collected during the wildfire event between Jan 7th to Jan 12th, 2025 were categorized as during the wildfire; collected 15 d after the fire between Jan 13th to Jan 27th, 2025 were categorized as post fire period.

We conducted quality control of the data by removing data from participants who wore the Oura Ring for less than 18 h, and further excluded heart rate and HRV data from participants with pacemakers.

### 2.3. Data analysis

Daily activity patterns and physiological stress markers are summarized using mean and standard deviation (SD). A paired t-test was used to compare activity patterns and physiological stress during versus before the wildfire and after versus before the fire. To leverage the longitudinal observations of the digital health markers related to activity patterns and physiological stress, we employed linear mixed-effects models to examine changes in these biosignals before, during, and after the fire. The linear mixed-effects models included a random intercept at the individual level, fixed effect dummy variables for fire stages (before, during, and after), and fixed effects variables for participants’ age, sex, race and ethnicity, and weekend and holiday indicators. We assessed whether participants received evacuation alerts based on their address before the fire and confirmed their evacuation status during fire events through surveys conducted after the fire. To assess the robustness of the model, we conducted sensitivity analysis including 1) additionally adjusting for outdoor measured daily PM_2.5_ levels, and 2) not adjusting temporal covariates including weekend and holiday indicators in the model.

All participants have answered surveys and provided evacuation details. A 2-tailed α less than 0.05 was considered statistically significant. To control for false discovery in multiple testing for the mixed-effects model analysis, we used the Benjamini–Hochberg (BH) procedure [20]. Data analyses were performed using R 4.3.3 with the lmerTest package from January to March 2025.

### 2.4. Ethical considerations

This study was approved by the IRB of the University of Southern California (#UP-23-01062) and all participants provided written informed consent at baseline for this wildfire study.

## 3. Results

**Figure 1.**
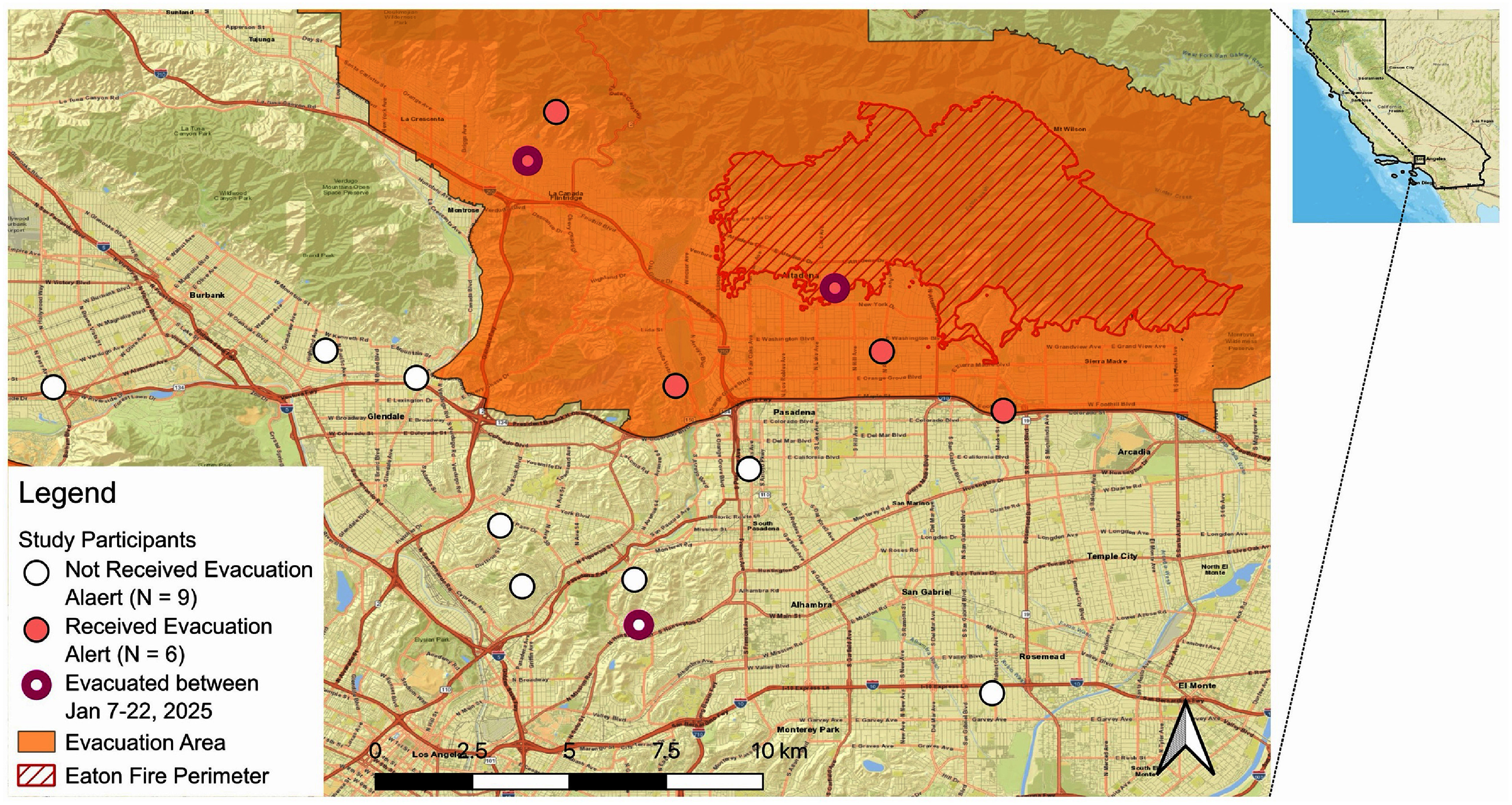
Map of study participants during 2025 Eaton Fire in Los Angeles. All participants, *N* = 15, age Mean ± SD: 73.2 ± 4.6 years; 8 (47%) male, 7 (53%) females; 10 (67%) white, 2(13%) Asian, and 3 (20%) Hispanic. The locations were shown for baseline (December 2024) and are intentionally altered to protect the privacy of participants. Basemap provided by ESRI.

A total of 21 participants were initially recruited from the on-going trial. Six participants completed the follow-up period before January 2025, and thus no digital health measurement in January 2025, end up with 15 older adults (mean age: 73.2 years, range: 67–82 years; 8 males and 7 females; 10 white, 2 Asian, and 3 Hispanics) included in the analysis. All fifteen participants lived within 10 miles of the Eaton Fire at baseline, and six received evacuation alerts; among them, two evacuated during the fire disaster (figure 1).

The mean durations were 18.9 d (range: 7–29), 3.8 d (range: 1–5), and 7.1 d (range: 2–14) per participant for wearable measurements before, during, and after the fire, respectively, totaling 437 person-days of measurements. Additionally, 50 person-day of HR and HRV measurements from 2 participants were removed due to having pacemakers, and *n* = 6 person-days of HR measurements were removed due to abnormally high value over 200 bpm. Figure S1 shows the flowchart of participant inclusion and data processing.

Table 1 presents the air pollution levels from the Central Los Angeles station of South Coast Air Quality Management District, as well as summary statistics of Oura Ring-measured activity patterns and physiological stress levels before, during, and after the wildfire periods. We observed a substantial increase in outdoor PM_2.5_ and PM_10_ air pollution, from 20.0 ± 1.2 and 35.4 ± 1.5 *µ*g m^−3^ to 55.2 ± 11.5 and 128.9 ± 15.3 *µ*g m^−3^ during the fire, and it decreased to 11.1 ± 1.7 and 37.8 ± 1.8 *µ*g m^−3^ after the fire. The results show that during the fire, sedentary time increased 8.5% from 563 ± 106–611 ± 78 min (*P*-value = .03), while total sleep time decreased from 381 ± 65–347 ± 38 min (*P*-value = .048). After the fire, we observed continued changes in both activity patterns and physiological stress measurements, including a 10.5% reduction in low-activity time from 283 ± 51–264 ± 55 min (*P*-value = .03), and a 2.6% increase in breath rate from 15.2 ± 1.2–15.6 ± 1.21 breath per minute (BPM) (*P*-value = .02). Figures S2–S9 in the supplementary information depict the individual-level data before, during and after the 2025 Los Angeles Wildfire event.

**Table 1.** Summary statistica of outdoor air pollution, activity patterns and physiological stress before, during and after the 2025 Los Angeles Eaton Fire.

	Before fire	During fire	After fire	*P*-value^a^	
	9th December 2024–6th January 2025 (4 weeks)	7th January 2025–11th January 2025 (5 d)	12th January 2025–27th January 2025 (2 weeks)	During vs Before	After vs Before
Outdoor PM_2.5_ (*µ*g m^−3^), Mean ± SD	20.0 ± 1.2	55.2 ± 11.5	11.1 ± 1.7	<0.001	<0.001
Outdoor PM_10_ (*µ*g m^−3^), Mean ± SD	35.4 ± 1.5	128.9 ± 15.3	37.8 ± 1.8	<0.001	<0.001
Outdoor Temperature (°C), Mean ± SD	13.8 ± 0.4	14.2 ± 0.6	12.6 ± 0.6	0.12	<0.001

Activity patterns					

Daily low activity time (min) Mean ± SD (range)	283 ± 51 (179–384)	279 ± 51 (163–356)	264 ± 55 (151–353)	0.58	0.03
Daily sedentary time (min) Mean ± SD (range)	563 ± 106 (401–783)	611 ± 78 (519–822)	520 ± 132 (329–769)	0.03	0.14
Daily total sleep duration (min) Mean ± SD (range)	381 ± 65 (236–506)	347 ± 38 (258–394)	366 ± 67 (253–503)	0.048	0.22
Daily steps (count) Mean ± SD (range)	7994 ± 1680 (4629–12154)	7614 ± 2064 (3687–10949)	7384 ± 1659 (3834–10828)	0.39	0.06

Physiological Stress					

Sleep-wake transition (times) Mean ± SD (range)	6.5 ± 1.9 (3.6–9.4)	5.4 ±2.5 (2.7–10.5)	6.7 ±2.9 (3.0–15.0)	0.26	0.49
Average sleep-period heart rate Mean ± SD (range)	62.9 ±5.9 (49.9–72.9)	62.5 ± 7.2 (49.3–73.8)	63.9 ± 6.0 (49.2–75.7)	0.95	0.08
Average sleep-period breath Mean ± SD (range)	15.2 ± 1.2 (12.7–17.8)	15.5 ± 1.4 (12.8–17.1)	15.6 ± 1.21 (13.5–17.9)	0.14	0.02
Average sleep-period HRV Mean ± SD (range)	30.2 ± 21. 8(13.3–81.4)	35.2 ± 22.0 (16.7–97.4)	34.8 ± 28.3 (14.3–110.4)	0.14	0.29

a Based on paired *t*-test.

**Figure 2.**
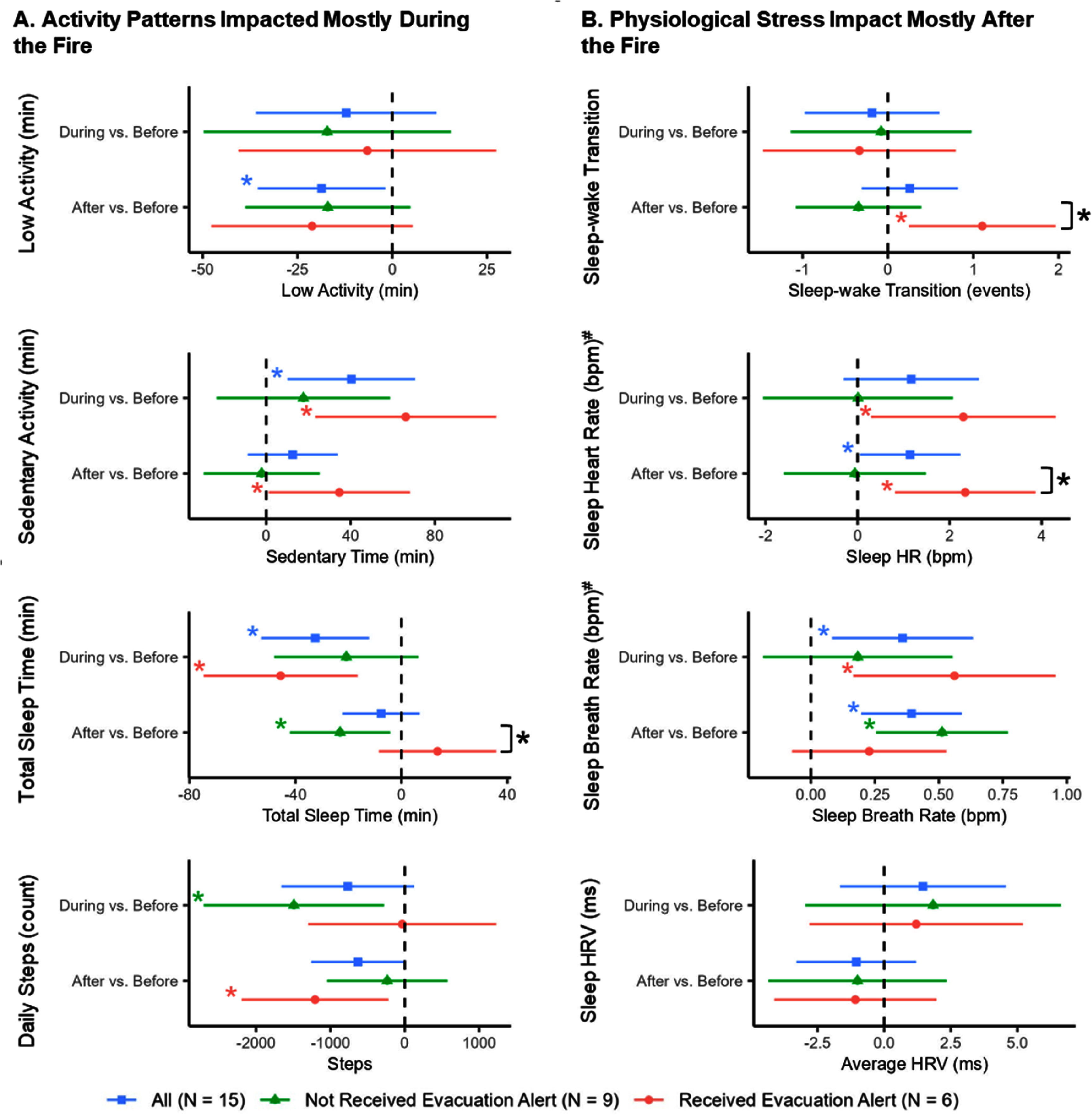
Change of physiological stress and activity patterns during and after the Eaton Fire for (A). Activity patterns changes during and after the fire compared to before and (B) Physiological stress changes during and after the fire. Changes are shown as point estimates with 95% confidence intervals. *P-value < 0.05 for main effect, *with bracket: *P*-value < 0.05 for interaction effects between participants who received evacuation versus not. #Two participants (who did not receive evacuation alerts) were removed due to using pacemakers that affect their heart rate.

Similar to the period-averaged statistics presented in table 1, the mixed-effects model based on daily data showed more substantial changes in activity patterns and physiological stress during and after the fire, compared to before fire. Figure 2 presents the mixed-effects analysis results for all participants, as well as the results separately for participants who received evacuation alerts (*n* = 6) and those who did not (*n* = 9). During the fire, we observed a 41 (95% confidence interval, CI: 11.3–71.2) minute decrease in sedentary time [*P*-value = .007, BH false discovery rate (FDR) = .04] and a 34 (95% CI: 12–55) minute decrease in total sleep time (*P*-value = .002, FDR = .016), as well as an 0.37 (95% CI: 0.09–0.64) BPM increase in sleep breath rate (*P*-value = .008, FDR = .038). Comparing to the baseline levels before wildfire, these changes correspond to 7.4% (95% CI: 2.0%–12.7%) increase in sedentary time, 8.9% (95% CI: 3.3%–14.5%) decrease of total sleep time, and 2.4% (0.6%–4.2%) increase in sleep breath rate (table S1 in supplementary material).

In participants receiving evacuation alerts, the changes were more significant, with a 67 (95% CI: 25–110) minute increase in sedentary time (*P*-value = .002, FDR = .016), a 48 (95% CI: 17–79) minute decrease in sleep duration (*P*-value = .002, FDR = .016), and a 0.58 (95% CI: 0.19–0.98) BPM increase in breath rate (*P*-value = .004, FDR = .027). Additionally, we found a significant reduction in daily steps [1424 (95% CI: 220–1424) steps, *P*-value = .021, FDR = .08] only among participants who did not receive evacuation alerts. Although physical activity and sleep duration disruption among study participants returned to baseline post-fire, elevated sleep heart rate [1.2 (95% CI: 0.06–2.2) BPM, *P*-value = .039, FDR = 0.11] and breath rate [0.4 (95% CI: 0.2–0.6) BPM, *P*-value < .001, FDR < .001] were observed after wildfire event. Comparing to baseline levels, these represent 1.8% (95% CI: 0.1%–3.6%) and 2.6% (95% CI: 1.3%—3.9%) higher physiological stress markers in sleep heart rate and breath rate. In addition, we found participants who received evacuation alerts shown stronger sustained physiological stress compared to whose who did not receive alerts after the wildfire. Notably, participants who received evacuation alerts exhibit higher increases in sleep heart rate [2.4 (95% CI: 0.9–3.9) BPM, *P*-value = .002, FDR = .016] and more fragmented sleep [1.2 (95% CI: 0.3–2.1) sleep-wake events, *P*-value = .008, FDR = .04] compared to participants who did not receive evacuation alerts (*P*-value for interaction = .03 and .01, respectively). Supplementary table S1 lists the numerical results of the daily-level analysis and the percentage changes of time-activity patterns and physiological stress during and after the fire compared to baseline.

Sensitivity analyses of the mixed effects model with additional adjusting for daily outdoor PM_2.5_ level and not adjusting temporal covariates (weekend and holiday indicators) shown similar results as the main model, indicating the robustness of the observed associations of activity patterns and physiological stress during and after wildfire among study participants (tables S2 and S3 in supplementary material).

## 4. Discussion

Wearables offer a valuable window into the real-time impact of WUI fires on activity patterns and physiological stress. Here, among a small number of older adults who resided close to the Eaton fire, we detected both immediate activity pattern changes and prolonged physiological stress following the Eaton fire that happened between 7 and 12 January 2025. Consistent with previous studies, our study indicated that physical activity and sleep duration decreased significantly during the fire [21, 22]. One study in San Francisco Bay Area found that during the wildfire smoke days, daily step count decreased 18% [21]. This is similar to our findings of 17.8% (1400 steps) reduction of daily steps among participants who did not receive evacuation alerts. However, we found there is no significant changes in daily steps among participants who received evacuation alerts. This implies potential differential activities patterns associated with wildfire emergency versus smoke. The percentage change of physiological stress after the fire compared to before the fire, including sleep heart rate and breath rate, is around 5%. Future investigations should assess whether the changes continued and understand the potential health effects. Our study found that physical activity and sleep time changes appear to recover quickly after the 2025 Los Angeles wildfire disaster, but physiological stress indicators such as sleep heart rate, breath rate, and sleep fragmentation remain elevated after the fire, especially among individuals affected by evacuation alerts. These sustained changes may be early markers of longer-term mental and physical health risks, providing insights into the mechanisms of wildfires.

Our study provided novel and important indentification of adverse physiological stress biomakers using digital wearables during and after the major 2025 Los Angeles wildfire, which can also help with indentification of the vulnerable populations after wildfire disasters and possible public health interventions to mitigate the adverse health effects [23]. However, this study has several limitations. First, the sample size is limited and only 15 older adults were included in the analysis, even though each participant contributed longitudinal daily observations. Due to study sample is small and only older adults were analyzed, therefore the generalization of our findings may be limited and warrant future studies to explore in other population and other wildfire events (such as Palisades Fire or other WUI fires). Second, we cannot distinguish the estimated associations from fire disasters versus smoke exposure, while all participants were living in areas close to Eaton Fire (within 15 km), where fire smoke and other air pollution was substantially increased during the fire for both indoor and outdoor environment [2–4]. Third, all participants are living close to the wildfire event and are all exposed, and we did not include a control group without exposure to Eaton Fire in this analysis. Due to these limitations, the findings of this study should be interpretated cautiously as the changes of activity patterns and physiological stress associated with during and after the Eaton Fire. Lastly, we did not collect the detailed relocation information of participants after evacuations, limiting our research on the influence of new displacement location on activity patterns and physiological stress. Larger studies utilizing digital wearables understanding the potential causal effects of the WUI fire events on activity patterns and physiological stress as well as predicting health outcomes following natural disasters are needed.

In conclusion, based on Oura Ring smart wearables, we found changes in activity patterns and increases in physiological stress, particularly among those in evacuation zones among a cohort of older adults during and after a 2025 Los Angeles Wildfire during 7–12 January 2025. Digital wearables can provide early indicators of adverse health outcomes following natural disasters.

## Data Availability

The data cannot be made publicly available upon publication because they contain sensitive personal information. The data that support the findings of this study are available upon reasonable request from the authors. Supplementary Tables & Figures available at https://doi.org/10.1088/2752-5309/ae7faf/data1.
